# Characterisation of Two Oxidosqualene Cyclases Responsible for Triterpenoid Biosynthesis in *Ilex asprella*

**DOI:** 10.3390/ijms16023564

**Published:** 2015-02-05

**Authors:** Xiasheng Zheng, Xiuxiu Luo, Guobing Ye, Ye Chen, Xiaoyu Ji, Lingling Wen, Yaping Xu, Hui Xu, Ruoting Zhan, Weiwen Chen

**Affiliations:** 1 Research Center of Chinese Herbal Resource Science and Engineering, Key Laboratory of Chinese Medicinal Resource from Lingnan, Guangzhou University of Chinese Medicine, Guangzhou 510006, China; E-Mails: zheng.x.s1987@163.com (X.Z.); lxx.carrot@163.com (X.L.); ygb900810@163.com (G.Y.); yavechan@163.com (Y.C.); xiaoyu3463@gmail.com (X.J.); 435892240wll@gmail.com (L.W.); yaping1965@163.com (Y.X.); zhanrt@gzucm.edu.cn (R.Z.); 2 The Key Laboratory of Technology of Breaking Cell Wall and Application in Chinese Medicine Decoction Pieces, Zhongshan Zhongzhi Pharmaceutical Group, Zhongshan 528437, China

**Keywords:** triterpene synthase, amyrin, *Ilex asprella*, gene cloning and expression

## Abstract

*Ilex asprella*, a plant widely used as a folk herbal drug in southern China, produces and stores a large amount of triterpenoid saponins, most of which are of the α-amyrin type. In this study, two oxidosqualene cyclase (OSC) cDNAs, *IaAS1* and *IaAS2*, were cloned from the *I. asprella* root. Functional characterisation was performed by heterologous expression in the yeast *Saccharomyces cerevisiae*. Analysis of the resulting products by gas chromatography (GC) and gas chromatography-mass spectrometry (GC-MS) showed that both genes encode a mixed amyrin synthase, producing α-amyrin and β-amyrin at different ratios. IaAS1, which mainly produces α-amyrin, is the second triterpene synthase so far identified in which the level of α-amyrin produced is ≥80% of total amyrin production. By contrast, IaAS2 mainly synthesises β-amyrin, with a yield of 95%. Gene expression patterns of these two amyrin synthases in roots and leaves of *I. asprella* were found to be consistent with the content patterns of total saponins. Finally, phylogenetic analysis and multiple sequence alignment of the two amyrin synthases against several known OSCs from other plants were conducted to further elucidate their evolutionary relationship.

## 1. Introduction

**Figure 1:**
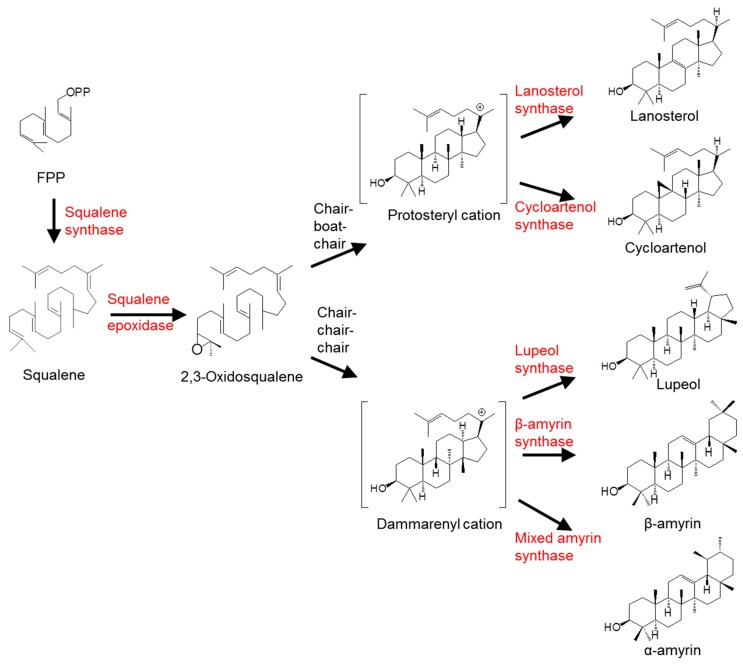
Biosynthesis of triterpenes catalysed by different types of oxidosqualene cyclase (OSC). Catalysed by a specific OSC, either protosteryl cation or dammarenyl cation could be formed according to different folding conformations of 2,3-oxidosqualene, leading to one or more final product(s).

In the plant kingdom, many species synthesise and store triterpenoids as a defense against microbes [1], insects [2] and herbivores [3], which is essential for their survival [4]. Triterpenoids are a diverse group of secondary metabolites that display various important medicinal properties [5], including anti-inflammatory [6], anti-tumour [7] and cholesterol-regulating [8] activities. Despite these important properties, drug development of triterpenoids is hampered by insufficient supply, caused by a low yield from herbal extraction. Therefore, a comprehensive understanding of the triterpenoid biosynthetic pathway is beneficial to meet the increasing demand [9]. Triterpenoid biosynthesis (see Figure 1) requires conjugation of two units of C15 to form squalene, the epoxidation of which produces the important substrate 2,3-oxidosqualene [10]. Furthermore, cyclisation of the 2,3-oxidosqualene, catalysed by oxidosqualene cyclases (OSCs), generates phytosterols or triterpenoids depending on the type of OSC involved [11]. In animals and fungi, 2,3-oxidosqualene is cyclised into sterol in the chair-boat-chair conformation, whereas in plants (especially higher plants), 2,3-oxidosqualene is cyclised through the chair-chair-chair conformation to form triterpenoids [12]. Hence, the cyclisation reaction catalysed by OSCs is considered a pivotal branch point for the biosynthesis of phytosterols and triterpenoids. Although information on the cyclisation mechanism remains limited, researchers believe that this process requires at least four steps: (1) Protonation of the 2,3-oxidosqualene; (2) A polyene addition cascade leading to the formation of specific cyclisation; (3) Shifts of hydride and/or methyl groups to prevent interfering side reactions; and (4) Deprotonation [11,13]. OSCs are the only enzymes responsible for the entire process described above.

Dozens of OSCs, which are named after their respective products (e.g., lanosterol synthases [14], lupeol synthases [15] and β-amyrin synthases (BASs) [16]), have been identified in the past decades. In addition, OSCs producing more than one specific compound, known as multifunctional OSCs, have also been identified in many plants. α-Amyrin and β-amyrin, which can be further transferred into ursane and oleanane, respectively, are the most common pentacyclic triterpenoid precursors in plants. Although many β-amyrin synthases (BASs) have been isolated and characterised, no enzyme producing α-amyrin as its sole product has been identified so far [17]. Multifunctional OSCs yielding α-amyrin could simultaneously synthesise one or more byproducts, such as β-amyrin, lupeol, or other triterpenes.

*Ilex asprella*, an Aquifoliaceae plant, is wide spread in southern China, especially in the Guangdong province. Due to its outstanding anti-inflammatory activity, it is commonly used in the treatment of flu and wounds [18]. The major chemical components of *I. asprella* are triterpenoid saponins, most of which are of the α-amyrin type. In our previous research, the transcriptome of *I. asprella* root was investigated, revealing a series of candidate genes (or fragments) likely to be involved in the biosynthesis of triterpenoid saponins, including nine candidate *OSC* genes [19]. The goal of this study was to clone and characterise two *OSC* genes from *I. asprella*. In addition, gene expression patterns of the two *OSC* genes in different tissues of *I. asprella* were analysed.

## 2. Results and Discussion

### 2.1. Gene Isolation and Functional Characterisation

#### 2.1.1. Isolation and Sequence Analysis of Two *IaAS* Genes

**Figure 2:**
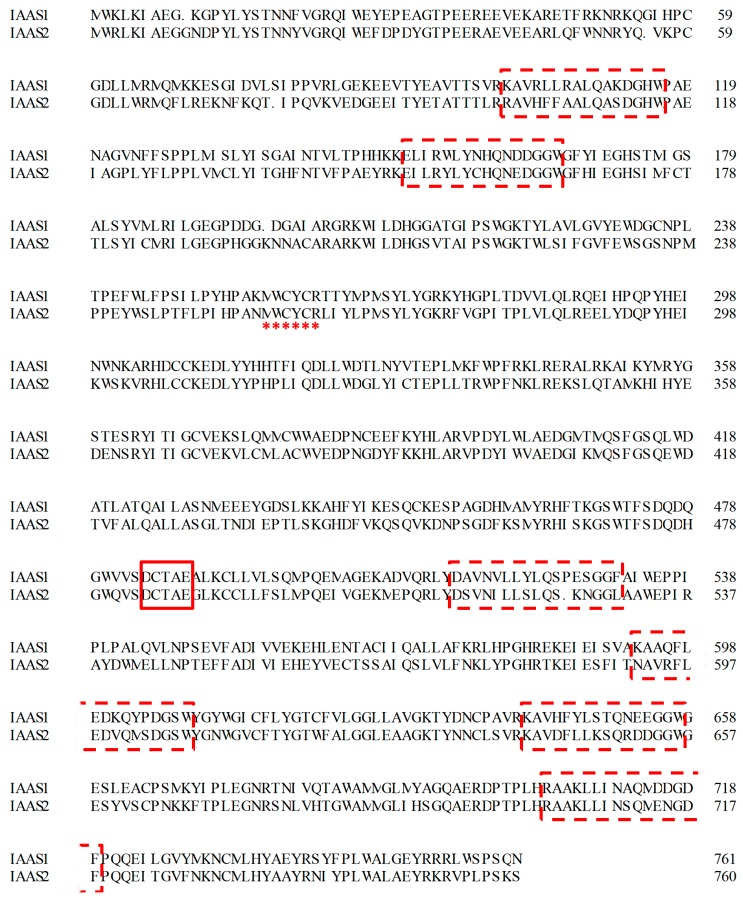
Amino acid sequences of *IaAS1* and *IaAS2*. The conserved QW motifs are highlighted by dashed line boxes, the DCTAE motif is emphasised by a solid line box and the MWCYCR motif is marked by asterisks.

Among the nine candidate *OSCs* identified in our previous study, there were two full-length cDNAs designated *IaAS1* and *IaAS2*. Two sets of primers were designed to amplify these sequences approximately 100 bp beyond the open reading frame (ORF) at either end of the cDNA. Both sequences were successfully isolated from the *I. asprella* root library and sequenced. The cDNA of *IaAS1* and *IaAS2* contain ORFs that encoding proteins of 762 and 761 amino acids with masses of 87.6 and 87.9 kDa, respectively. *IaAS1* and *IaAS2* exhibit 62% identity to each other in protein sequence. In addition, *IaAS1* shares 82% sequence similarity with mixed amyrin synthase (*Catharanthus* *roseus*, AFJ19235.1), whereas *IaAS2* is 86% identical to β-amyrin synthase (*Vitis* *vinifera*, XP_002270934.1). As shown in Figure 2, six QW motifs [(K/R)(G/A)XX(F/Y/W)(L/I/V)XXXQXXXGXW] were found in the protein sequences of both *IaAS1* and *IaAS2*, which are believed to be responsible for strengthening the structure of the enzyme and stabilising its carbocation intermediates, as well as a DCTAE motif, which might play an important role in substrate binding [20,21]. Furthermore, a MWCYCR motif was identified that is believed to be related to the product specificity of BAS [22].

#### 2.1.2. Gene Cloning and Protein Expression

**Figure 3:**
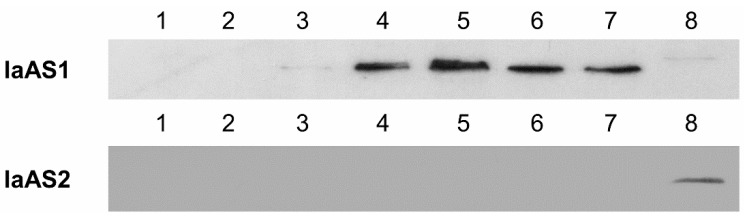
Western blot analysis of IaAS1 and IaAS2 expression in yeast transformants. 1: Negative control (pYES-DEST 52), 2: 0 h, 3: 4 h, 4: 8 h, 5: 16 h, 6: 24 h, 7: 32 h after induction, 8: Positive control (pEXPR-gus).

To elucidate the enzymatic activities of *IaAS1* and *IaAS2*, the core sequences of the two genes were cloned into the expression vector pYES-DEST52 under the control of galactose (GAL) promoter with a 6× His-tag modification and transformed into *S. cerevisiae* INVSc1, which synthesises 2,3-oxidosqualene endogenously. The equivalent of two OD_600_ of yeast transformants were harvested at six different time points during 32 h of induction by Gal. Western blot analysis of the total protein extracted from the cells showed that IaAS1 was successfully expressed during the 32 h induction, with a maximum band intensity observed at 16 h (see Figure 3). However, no IaAS2 protein was detected (see Figure 3). This indicated that the expression level of IaAS1 was significantly higher than that of IaAS2, which might have been too low to be detected.

#### 2.1.3. Functional Analysis of IaAS1 and IaAS2 in Yeast

**Figure 4:**
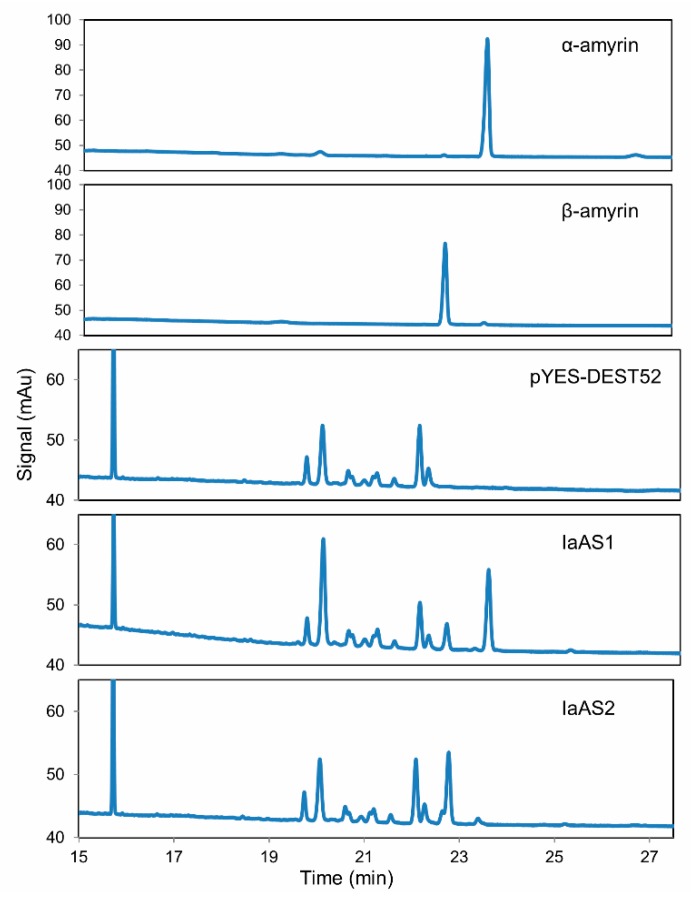
Gas chromatography analysis of the products of IaAS1 and IaAS2 over-expressed in yeast. pYES-DEST52 was served as a negative control and α-amyrin and β-amyrin as standards.

**Figure 5:**
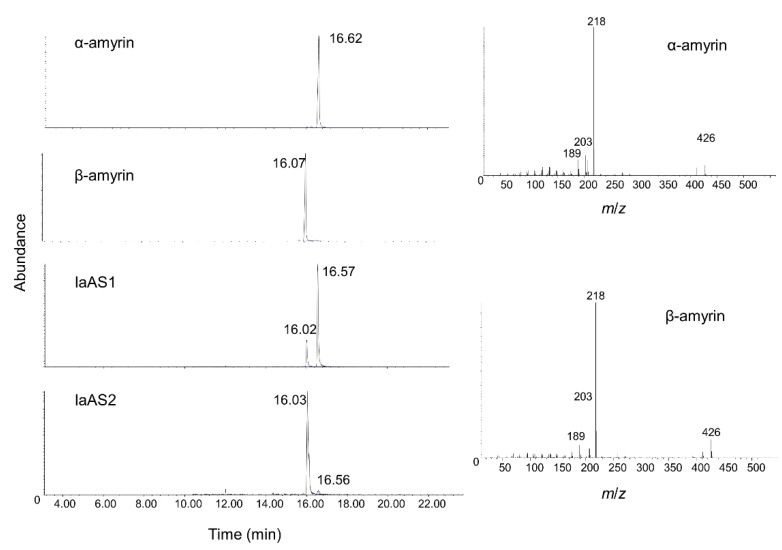
GC-MS (with an Electron Impact source) analysis of transient IaAS1 and IaAS2 expression. Products were monitored on the basis of the intensity of the base peak (*m*/*z* 218), with α-amyrin and β-amyrin as standards. Two peaks identified in both IaAS1 and IaAS2 exhibit the same retention time and MS fragments as the standards.

Triterpene products were extracted after 72 h of induction and analysed using gas chromatography (GC). Both IaAS1 and IaAS2 extracts contain two compounds that were not present in the control cells carrying empty vector (see Figure 4). To identify the products of IaAS1 and IaAS2, the cell extracts were submitted to gas chromatography-mass spectrometry (GC-MS) analysis. Two compounds found in IaAS1 and IaAS2 extracts were identified as α-amyrin and β-amyrin, respectively, by comparing their retention times and mass fragment patterns with an authentic standard, indicating that both enzymes are mixed ASs (see Figure 5). α-Amyrin was the major product of IaAS1, with a 4:1 ratio to β-amyrin, whereas β-amyrin was the main product of IaAS2, with a ratio of 19:1 to α-amyrin. Compared with other mixed ASs, IaAS1 exhibits a unique product specificity, with α-amyrin accounting for up to 80% of the enzyme product, second only to MdOSC1 in *Malus × domestica* [23].

### 2.2. Gene Expression and Chemical Content Patterns

#### 2.2.1. Expression Patterns of the *IaAS* Genes in Different Tissues of *I. asprella*

**Figure 6:**
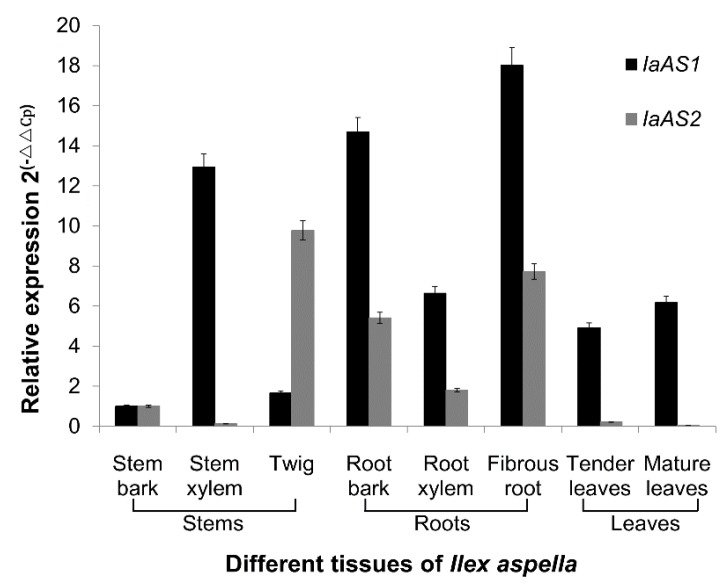
Expression patterns of *IaAS1* and *IaAS2*. Relative expression levels of both target genes in different tissues were normalised to the reference gene, *β-tubulin*, and further evaluated relative to the stem bark sample. Error bars represent the standard errors of the means calculated from three technical replicates.

To assay the expression patterns of both *AS* genes, real-time quantitative polymerase chain reaction (RT-qPCR) was performed on eight different tissues of* I. asprella*, including stem bark, stem xylem, twig, root bark, root xylem, fibrous roots, tender leaves and mature leaves, with *β-tubulin* as a reference gene. As shown in Figure 6, relatively higher expression levels were detected for both genes in the roots than in the stems and leaves, indicating that roots might be the primary location of triterpenoid synthesis in *I*. *asprella*. *IaAS2* exhibits a lower expression level than *IaAS1* in all tissues except for the twig. In addition, the gene expression levels observed for both *AS* genes were in agreement with those observed in the *I*. *asprella* root transcriptome characterised in our previous study. Of the nine candidate *OSCs* identified,* IaAS1* was expressed at the highest level (with a FPKM value of 163.4450; FPKM represent for Fragments per kilobase of exon model per million mapped fragments), whereas *IaAS2* was expressed at an overall low level (with a FPKM value of 1.7797), indicating that *IaAS1* might be the most active triterpene synthase in *I. asprella*.

#### 2.2.2. Localisation Patterns of Triterpenoid Saponins

**Figure 7:**
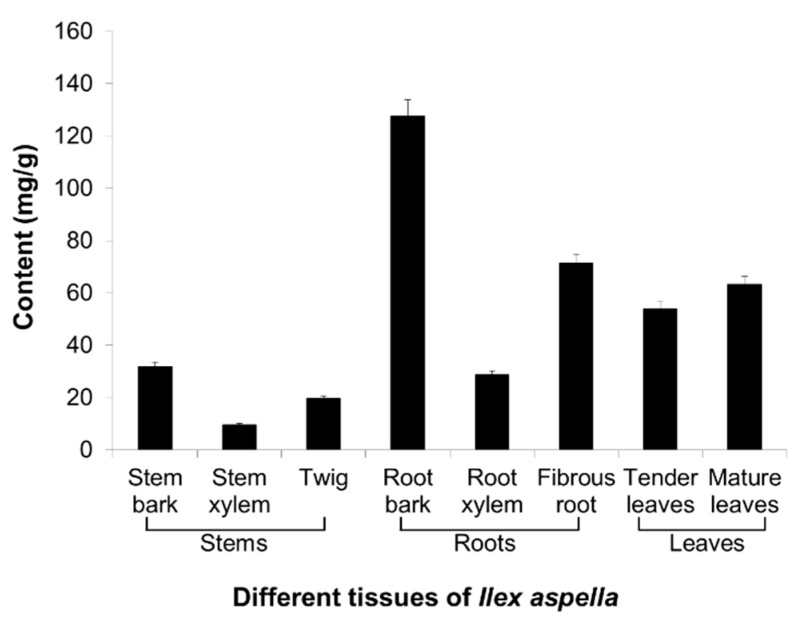
Localisation patterns of total saponin in different tissues of *I. asprella.* Absolute content of total saponins in different tissues were calculated according to a standard curve of a gradient concentration of ursolic acid solution. Error bars represent the standard errors of the means calculated from three technical replicates.

Chemical analysis was carried out by ultraviolet spectrophotometry after coloration of extracts from the plant tissues listed above. Results (see Figure 7) showed that the total saponin content of roots was higher than that of leaves and stems, indicating that roots might be the major site of triterpenoid saponin storage. The pattern of triterpenoid saponin localisation in roots and leaves were similar to the gene expression patterns of the two *ASs* described in this study, whereas stems exhibited high gene expression levels, but low triterpenoid saponin content. In summary, triterpenoid saponins are synthesised in all tissues of *I. asprella* but are stored in the roots and leaves.

### 2.3. Discussion

*I. asprella* contains mainly ursane-type triterpenoid saponins. Nine candidate *OSC* genes were identified in the *I. asprella* root transcriptome. It was necessary to find out which candidate(s) is/are responsible for the biosynthesis of saponins. Our results demonstrated that *IaAS1*, which was expressed at the highest level of the candidate *OSCs* and whose major product is α-amyrin, must play an important role in the biosynthetic process. Although the expression of *IaAS2* gene was quite low in *I. asprella*, the gene is fully functional in yeast. Therefore, the biosynthesis of triterpenoid saponins in *I. asprella* must be regulated at the gene expression level.

#### 2.3.1. Phylogenetic Analysis of the IaAS Proteins and Other Known OSCs

**Figure 8:**
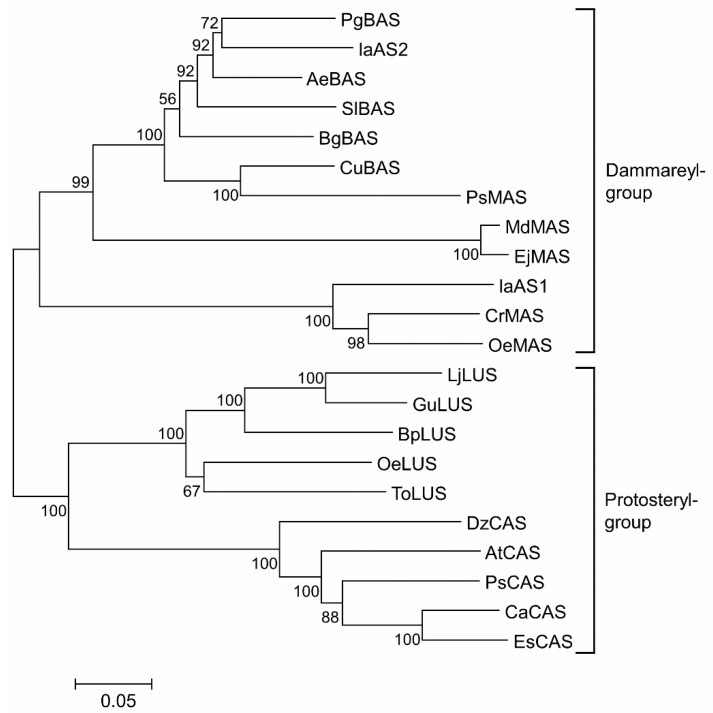
Bootstrapped neighbour-joining tree of known OSCs. The tree was constructed by MEGA 5.1 software using the neighbour-joining method, with an evaluation method of Bootstrap for 1000 times. AeBAS: *Aralia elata* (GenBank accession number: ADK12003.1), AtCAS: *Arabidopsis thaliana* (NP_178722.1), BgBAS: *Bruguiera gymnorhiza* (BAF80443.1), BpLUS: *Betula platyphylla* (BAB83087.1), CaCAS: *Centella asiatica* (AAS01524.1), CrMAS: *Catharanthus roseus* (AFJ19235.1), DzCAS: *Dioscorea zingiberensis* (CAM91422.1), EjMAS: *Eriobotrya japonica* (AFP95334.2), EsCAS: *Eleutherococcus senticosus* (AFC67276.1), GuBAS: *Glycyrrhiza uralensis* (ACV21067.1), GuLUS: *G. uralensis* (BAL41371.1), LjLUS: *Lotus japonicus* (BAE53430.1), MdMAS: *Malus domestica* (ACM89977.1), OeLUS: *Olea europaea* (BAA86930.1), OeMAS: *O. europaea* (BAF63702.1), PqBAS: *Panax quinquefolius* (AGG09939.1), PsCAS: *Pisum sativum* (BAA23533.1), PsMAS: *P. sativum* (BAA97559.1), SlBAS: *Solanum lycopersicum* (NP_001234604.1), ToLUS: *Taraxacum officinale* (BAA86932.1). MAS is for mixed amyrin synthase, while BAS is for β-amyrin synthases, LUS is for lupeol synthases and CAS is for cycloartenol synthases.

To extend our knowledge of the two ASs identified in this study, a phylogenetic tree was generated on the basis of the deduced amino acid sequences of plant OSCs with known function (as shown in Figure 8). These OSCs were grouped into two main branches, representing two types of functional products (protosteryl derivates and dammarenyl derivates). In the protosteryl group, lupeol synthases and cycloartenol synthases form two distinguished clades, whereas the dammarenyl group, β-amyrin synthases were intercrossed with mixed amyrin synthases. IaAS1 was grouped with the mixed amyrin synthases, which is consistent with our result described above, reporting heterologous expression. Yet, IaAS2 and the mixed amyrin synthase from *Pisum* *sativum* (BAA97559.1) exhibit a closer relationship to the β-amyrin synthases rather than to the mixed amyrin synthases, which synthesise multiple products, including β-amyrin. Thus, a more in-depth examination of the sequences of these OSCs would contribute to understanding the relationships between them.

#### 2.3.2. Unique Residues and the Catalytic Specificities of Different OSCs

In addition to creating a phylogenetic tree, the protein sequences of these OSCs were subjected to multiple alignment analysis. Unique residues were distinguished and are listed in Table 1. The aromatic residues Trp257 and Tyr259 (corresponding to Trp259 and Tyr261, respectively, in *Panax ginseng* BAS [12]) for the MWCYCR motif (aa 256–261) are believed to be crucial for β-amyrin specificity [23]. Substitution of Trp257 with a leucine residue might result in different product specificity for lupeol. Meanwhile, exchange of Tyr259 for a histidine residue might be responsible for the biosynthesis of cycloartenol. MASs, BASs and LUSs all have two important residues (SerPhe) in the MQSFGSQ motif (aa 409–416), which correspond to their catalytic differences; whereas those residues in CASs were altered to three residues (GlyTyrAsn). Moreover, MASs contain several unique residues at various positions not found in the other three synthases, including Ser (or Pro) 137, Thr263, Tyr (or Gln) 354, Ser (or Pro) 373, Gln (or Met) 375, Leu (or Val) 617, Thr677 and Asp715. The sequence characteristics of these OSCs provide possible explanations for their relationships in the phylogenetic tree described above.

**Table 1:** Comparison of special sites in the amino acid sequences of 22 known OSCs.

OSCs	137	254	256–261	263	317	354	373	375	409–416	614	617	677	715
IaAS1	S	A	MWCYCR	T	T	Y	S	Q	MQSF-GSQ	I	L	T	D
CrMAS	S	A	MWCYCR	T	S	Y	S	Q	MQSF-GSQ	I	L	T	D
EjMAS	P	S	MFCYCR	T	G	Q	P	M	MQSF-GSQ	I	V	T	D
MdMAS	P	S	MFCYCR	T	G	Q	P	M	MQSF-GSQ	I	V	T	D
OeMAS	S	A	MWCYCR	T	S	Y	S	Q	MQSF-GSQ	I	L	T	D
PsMAS	T	A	MLCYCR	V	P	H	V	C	LHSF-GSQ	I	T	S	E
IaAS2	T	A	MWCYCR	I	P	H	V	C	MQSF-GSQ	V	T	S	E
AeBAS	T	A	MWCYCR	V	P	H	V	C	MQSF-GSQ	V	T	S	E
BgBAS	T	A	MWCYCR	V	P	H	V	C	MQSF-GSQ	V	T	S	E
CuBAS	T	A	MWCYCR	V	P	H	V	C	MQSF-GSQ	V	T	S	E
PgBAS	T	A	MWCYCR	V	P	H	V	C	MQSF-GSQ	V	T	S	E
SlBAS	T	A	MWCYCR	V	P	H	V	C	MQSF-GSQ	V	T	S	E
BpLUS	T	G	ILCYSR	V	P	H	V	C	MQSF-GCQ	V	I	S	E
GuLUS	T	G	MLCYCR	V	P	H	V	C	IQSF-GCQ	I	T	A	E
LjLUS	T	G	MLCYCR	V	P	H	V	Y	IQSF-GSQ	I	T	A	D
OeLUS	T	G	MLCYCR	V	P	H	V	C	MQSF-GCQ	I	T	S	E
ToLUS	T	G	MLCYCR	V	P	H	V	C	MQSF-GCQ	I	T	S	E
AtCAS	T	G	MWCHCR	V	P	H	V	N	MQGYNGSQ	V	T	S	E
CaCAS	T	G	MWCHCR	V	P	H	V	N	MQGYNGSQ	V	T	S	E
DzCAS	T	G	MWCHCR	V	P	H	V	N	MQGYNGSQ	V	T	P	E
EsCAS	T	G	MWCHCR	V	P	H	V	N	MQGYNGSQ	V	T	S	E
PsCAS	T	G	MWCHCR	V	P	H	V	N	MQGYNGSQ	V	T	S	E

Although the enzymatic mechanism of OSCs remains to be elucidated, it has been hypothesised that their product specificity depends on differential rearrangement and deprotonation of the intermediates, which are guided by residues in the active site [24]. Thus, the distinguishing residues presented above might determine the catalytic specificity of MASs, which remains to be authenticated by experiments such as site-directed mutagenesis or domain swapping. Meanwhile, those specific residues might demonstrate a developing molecular evolution of OSCs, which is supported by the diverse specificities of OSCs in different species and by the coexistence of multiple *OSC* genes in one species. Researchers have suggested that LUS and BAS evolved from a common ancestor, CAS [25]. It has been hypothesised that an increasing evolution in MAS might arise from BAS. In the nine candidate OSCs identified in *I. asprella* root, only two were characterised in this study. Characterisation of the other seven candidates will help to more systematically elucidate the evolutionary relationship of OSCs in *I. asprella*.

## 3. Experimental Section

### 3.1. Materials

Three-year-old potted *I. asprella* were kindly provided by Nanling Pharmaceutical Co. Lit. *Escherichia coli* DH 5α (Invitrogen, Carlsbad, CA, USA) and *S*. *cerevisiae* INVSc1 (Invitrogen) were stored and cultivated in our laboratory. α-Amyrin and β-amyrin of 98.5% purity were purchased from Sigma-Aldrich (Shanghai, China). Ursolic acid of 98.0% purity was obtained from Guangdong Institute for Food and Drug Control (Guangzhou, China). Other enzymes, unless otherwise specified, were purchased from TAKARA (Otsu City, Japan).

*3.2. cDNA Preparation and Cloning of AS* *Genes*

Total RNA was extracted from the roots of *I. asprella* using RNAiso Plus (TAKARA) and Fruit-mate™ (TAKARA) for RNA Purification. Then, polyadenylated RNA was isolated and translated into cDNA using oligo(dT) primers by following the protocol of the PrimeScript™ RT-PCR Kit (TAKARA). The cDNA served as a template for amplifying the core sequences of *IaAS1* and *IaAS2* using high fidelity DNA polymerase with two sets of gene-specific primers (IaAS1-F and IaAS1-R, and IaAS2-F and IaAS2-R; see Table 2) under the following cycling conditions: 98 °C for 2 min; 30 cycles of 98 °C for 10 s, 50 °C for 15 s, 72 °C for 15 s; and 72 °C for 5 min. The resulting PCR products, following the addition of an A-tail, were ligated into the pGEM-T easy vector (Promega, Madison, WI, USA), transformed into DH 5α *E. coli* and submitted for sequencing.

**Table 2:** List of primers used in this study.

Primer ID	Primer Sequence (5'→3')	Length (bp)
IaAS1-F	TCTCTCTGTGTTTATGGGTA	20
IaAS1-R	GAACACTGAAGGATACAAAC	20
IaAS2-F	GCCACAGTTATCTTCGTATT	20
IaAS2-R	CATACTTCAAGGACCTCAAA	20
attB1-IaAS1	AAAAAGCAGGCTTCATGTGGAAGCTTAAGATTGC	34
attB2-IaAS1	AGAAAGCTGGGTCGACATTCTGGGAAGGTGACC	33
attB1-IaAS2	AAAAAGCAGGCTTCATGTGGAGGCTGAAGATTGC	34
attB2-IaAS2	AGAAAGCTGGGTCCCACAGGCTTTTTGATGG	31
attB1	GGGGACAAGTTTGTACAAAAAAGCAGGCT	29
attB2	GGGGACCACTTTGTACAAGAAAGCTGGGT	29
RTIaAS1-F	GTACGCTGGACAGGCTGAGAG	21
RTIaAS1-R	CGCCTACGGTATTCACCAAGT	21
RTIaAS2-F	GCATGGGAGCCAACAGGAG	19
RTIaAS2-R	TCTTCGAGGAACCGAACAGC	20
TUA-F	TATCGCCAGCTTTTCCATCC	20
TUA-R	CCACCACCAACAGCACTAAAC	21

### 3.3. Construction of Expression Vectors and Yeast Expression

Gateway cloning technology was applied in the construction of expression vectors in this study. pGEM-T easy vectors (Promega) containing the AS core sequences were used as templates for the first round of PCR. PCR amplification using inner primers (attB1-IaAS1, attB2-IaAS1, attB1-IaAS2, attB2-IaAS2; see Table 2) under the following temperature procedure: 98 °C for 2 min; 10 cycles of 98 °C for 10 s, 64 °C for 15 s, 72 °C for 15 s. Next, a set of outer adaptors (attB1 and attB2; see Table 2) were added to the products of the first round of PCR, along with supplementary DNA polymerase. The second round PCR reaction was performed under the following conditions: 98 °C for 2 min; 5 cycles of 98 °C for 10 s, 45 °C for 15 s; 20 cycles of 98 °C for 10 s, 64 °C for 15 s, 72 °C for 15 s; and 72 °C for 5 min.

Next, a BP reaction was carried out by mixing the attB-tailing AS with pDONR vector, catalysed by BP clonase (Invitrogen) to generate pENTR vector containing the target gene in a proper reading frame. The resulting pENTR vectors were transformed into DH 5α* E. coli* and then submitted to sequencing to select vectors positive for fusion products. pENTR vectors were then used for the LR reaction, together with the pYES-DEST52 vector (Invitrogen), to generate the expression vectors pEXPR-IaAS1 and pEXPR-IaAS2, which could be translated into proteins with a V5 antigen epitope and a 6× His tag. The pEXPR-gus vector, resulting from a pENTR-gus provided with the LR clonase Kit (Invitrogen), served as a positive control. Finally, *S. cerevisia* strain INVSc1 were transformed with pEXPR-IaAS1 and pEXPR-IaAS2 using a standard lithium acetate protocol [26], with pYES-DEST52 as the negative control and pEXPR-gus as the positive control.

### 3.4. Protein Expression

After overnight growth in SC-U media containing 2% raffinose, transformant yeast was isolated and resuspended in SC-U media containing 2% galactose for inductive cultivation. An equivalent of two OD_600_ cells were harvested at different time points over a period of 32 h and extracted for total protein. V5 mouse monoclonal antibody (Invitrogen) was used to identify target proteins with a V5 epitope by Western blotting.

### 3.5. GC and GC-MS Analysis

Yeast cells incubated in induction media for 72 h were collected and refluxed in 20% KOH/50% EtOH for 30 min, then extracted with hexane. Extracts were evaporated to dryness, resuspended in methanol and submitted to GC and GC-MS analysis directly. GC analysis was performed on an Agilent 7890B GC machine equipped with a flame ionisation detector (FID) and an HP-5MS column (0.25 mm × 30 m × 0.25 μm, Agilent, Santa Clara, CA, USA). The column temperature was set at 80 °C for 1 min, followed by a 20 °C/min ramp to 310 °C, held at 310 °C for 15 min. Injector and detector temperatures were both set at 250 °C. The sample was injected into a splitless injection mode and the carrier gas was helium with a flow rate of 1.2 mL/min. GC-MS was performed on a 6890C GC machine with a HP5973MSD mass spectrometer and a DB-5MS column (0.25 mm × 30 m × 0.25 μm, Agilent). The column temperature was initially held at 80 °C for 1 min then raised to 280 °C at a rate of 10 °C/min, and held at 280 °C for 3 min. The injector was set at a 10:1 split stream mode, with a temperature of 250 °C. The flow rate of helium carrier gas was 0.7 mL/min. Ionisation of samples was performed by electron impact at 70 eV to estimate the chemical structure, with a mass range acquired over *m*/*z* 29–500. The ion source temperature was held at 230 °C and the GC transfer line at 280 °C. For quantification, samples were run in selected ion mode, detecting ions 218 and 426.

### 3.6. RT-qPCR Analysis

Total RNA was isolated from different tissues of *I. asprella* and was reverse transcribed to cDNA with an equivalent amount of 1 μg total RNA for each sample as described above in three biological replicates. qPCR reactions were carried out on a CFX96 machine (Bio-Rad, Hercules, CA, USA). Reactions were performed in triplicate with 2 μL four-fold diluted cDNA, 10 μL SsoFast Eva Green Supermix (Bio-Rad) and 0.5 μL gene specific primers (RTIaAS1-F and RTIaAS1-R, and RTIaAS2-F and RTIaAS2-R; see Table 2) to a final volume of 20 μL, and run under the following conditions: 94 °C for 30 s; 40 cycles of 94 °C for 5 s, 60 °C for 5 s; and a raise from 65–95 °C at a rate of 5 °C/s. A negative control of distilled water was included in each run.

### 3.7. Chemical Analysis of Total Saponin Content

One gram of plant tissue was dried, crushed into a fine powder and extracted in 20-fold 70% ethanol for 30 min with ultrasonic treatment, filtered and metered to a volume of 50 mL. One hundred microliters of extract was removed and dried by evaporation. Then, 0.4 mL vanillin-glacial acetic acid and 1.6 mL perchloric acid were added, incubated at 60 °C for 15 min, then cooled at 4 °C for 2 min, and diluted to a volume of 10 mL. An equivalent volume of coloured extracts were detected at an absorbance of 545 nm and quantitated with a standard curve established by detection of a series of concentrations of standard ursolic acid, according to the method described by Uematsu* et al.* [27] and Zhao* et al.* [28]. The same procedure was followed for distilled water to establish a blank control.

## 4. Conclusions

To date, dozens of triterpenoid saponins have been isolated from *I. asprella* roots and leaves and characterised. Their chemical distribution patterns are supported by the discovery of several OSCs and their product specificities in the organism. Information describing the relationship between gene expression and terpenoid biosynthesis is limited, yet worth exploring. In addition, identification of candidate P450s and UTGs responsible for the downstream biosynthesis of triterpenoid saponins in *I. asprella* is imperative. Moreover, elicitor-induced expression regulation* in vitro* or cultivated *I. asprella* would be beneficial for better understanding the contribution of these biosynthetic enzymes. In our study, a rough picture of chemical and gene expression patterns in *I. asprella* was presented. A more thorough analysis of the remaining paralogous OSCs and the metabolomics data will help build a clear image of triterpenoid saponin biosynthesis of *I. asprella*.
